# Cell-Free DNA, Tumor Molecular Concordance, and Clinical Correlates of Patients with Cancer Treated in a Large Community Health Care Network

**DOI:** 10.1016/j.jmoldx.2025.05.007

**Published:** 2025-06-25

**Authors:** William A. LaFramboise, Patti Petrosko, Phillip H. Gallo, Louis Gil, Tuong L. Lam, Robin M. Barr, Philip E. Schumacher, Harmeet K. Kharoud, Katherine M. Taylor, Emily Dalton, Bella Bapat, Sefali Patel, John Nakayama, Christie J. Hilton, Lisa B. Ercolano, Ali H. Zaidi, Casey J. Allen, Thomas Rachman, Oana Carja, Russell Schwartz, Patrick L. Wagner, David L. Bartlett

**Affiliations:** ∗ Allegheny Health Network Cancer Institute, Pittsburgh, Pennsylvania; † Illumina, Inc., San Diego, California; ‡ Ray and Stephanie Lane Computational Biology Department, Carnegie Mellon University, Pittsburgh, Pennsylvania; § Department of Biological Sciences, Carnegie Mellon University, Pittsburgh, Pennsylvania

## Abstract

Blood collection, plasma processing, and cell-free DNA (cfDNA) purification were optimized to capture circulating tumor DNA without blood cell background DNA among 874 patients with cancer. cfDNA comprised predominantly mononucleosomal fragments [*n* = 874; mean ($\bar{\text{x}}$) ± SD = 166 ± 5 bp] that generated comparably sized sequencing reads ($\bar{\text{x}}$ ± SD = 162 ± 25 bp). Despite a vast range of cfDNA concentrations (0.50 to 1132.9 ng/mL) across 21 tumor types, matched tumor and blood specimens (*n* = 430 patients) revealed high concordance for coding (median = 97%) and clinical oncogenic mutations (median = 88% concordance). Therapeutically actionable mutations were identified in 233 patients by both assays, whereas 126 patients had oncogenic mutations without an established pharmacotherapeutic agent. An additional 48 patients (11%) had actionable mutations detected only in cfDNA assays, whereas 23 patients (5%) had mutations in tumor only. Concordance was high in both prevalent (lung, breast, and colon) and rare tumors (appendiceal, sarcoma). Cell-free DNA levels from diagnostic blood specimens were a strong indicator of patient survival duration independent of age, sex, tumor type, and stage, demonstrative of a potentially important role as a prognostic biomarker. Mutations in established oncogenes and tumor suppressors were readily detectable across all tumor types in circulating tumor DNA, indicating a diagnostic role for cfDNA from blood extending beyond the identification of companion therapeutics to patient screening and monitoring.

Comprehensive genomic profiling (CGP) of solid tumors using next-generation sequencing (NGS) has become a standard of care for patients with cancer to obtain diagnostic information, identify biomarkers for growing numbers of targeted therapies, follow guideline-endorsed use of US Food and Drug Administration–approved drugs, or select among extensive clinical trials for those applicable to individual patients. Clinical CGP assays evolved largely through adaptation of NGS methods to interrogate short DNA fragments typical of formalin-fixed, paraffin-embedded (FFPE), pathology tumor specimens incorporating an enrichment process for target cancer genes or hot spot domains using hybridization-capture enrichment or amplicon PCR, respectively.^1^^,^^2^ Improved methods to collect, isolate, and analyze the small fragments of circulating tumor DNA (ctDNA) found in plasma led to its adoption as an alternate clinical substrate for detection of cancer biomarkers when tumor tissue was unavailable, or when biopsy was contraindicated.^3^ Most US Food and Drug Administration–approved clinical assays for interrogating ctDNA for diagnostic purposes have relied on the same enrichment techniques and NGS sequencing platforms developed for tumor tissue diagnosis but with specialized blood collection and deep sequencing protocols focused on smaller targeted panels to detect ctDNA fragments found at low concentrations in the cell-free DNA (cfDNA) compartment of plasma.

National Comprehensive Cancer Network policy guidelines indicate that blood samples should not replace tumor tissue as the standard for diagnosis and treatment of patients with cancer. However, National Comprehensive Cancer Network policy recognizes the use of ctDNA when tumor tissue is inaccessible to identify a targeted drug therapy for specific gene mutations [eg, non–small-cell lung cancer, *https://www.nccn.org/professionals/physician_gls/pdf/nscl.pdf* (registration required); stage IV colorectal cancer, *https://www.nccn.org/professionals/physician_gls/pdf/colon.pdf* (registration required); hormone receptor–positive/human epidermal growth factor receptor 2–negative (HR^+^/HER2^–^) breast cancer, *https://www.nccn.org/professionals/physician_gls/pdf/breast.pdf* (registration required); metastatic pancreatic cancer, *https://www.nccn.org/professionals/physician_gls/pdf/pancreatic.pdf* (registration required); and metastatic prostate cancer, *https://www.nccn.org/professionals/physician_gls/pdf/prostate.pdf* (registration required), all last accessed February 18, 2025]. Academic and commercial ctDNA assays using NGS technology demonstrated high sensitivity and concordance in validation studies compared with tumors or orthogonally validated plasma samples in the identification of critical variants associated with targeted therapies.^4^, ^5^, ^6^, ^7^, ^8^, ^9^ However, the clinical utility obtained using these tests for diagnosis and management of patients in the health care setting has resulted in widely varying performance characteristics rarely approaching the performance standards established in validation studies. A study of 129 key cancer genes across 617 patients performed within the Memorial Sloan Kettering Cancer Center demonstrated overlap of 59% of mutations detected between their tumor and blood-based assays.^4^ A recent study of four cancer types from 3209 patients tested at a commercial laboratory (Tempus, Chicago, IL) reported a concordance rate of 66.4% for actionable variants comparing matched tissue and ctDNA results.^10^ In a similar pan cancer cohort using different commercial assays (tissue: Foundation Medicine, Boston, MA; ctDNA: Guardant 360, Redwood City, CA), 57.5% of the patients exhibited no mutual gene alterations between tests.^11^ Similar findings were observed in clinical studies of patients with stage IV non–small-cell lung cancer evaluating ctDNA results (Guardant 360) paired to NGS tissue testing (Oncomine Focus Assay; ThermoFisher, Waltham, MA) where the ctDNA test showed a sensitivity of 67.7% compared with the tissue assay.^12^ Although demand for diagnostic liquid biopsy testing continues to expand, defining the broad spectrum of variables impacting performance of ctDNA tests upon deployment for clinical application could improve their overall effectiveness and utility as a comparable or complementary test to tumor tissue testing.

A major hurdle in developing and optimizing concordant tumor and blood-based CGP assays is the limited availability of paired tumor and blood specimens with precisely characterized collection times, treatment status, tumor stage, and preanalytical processing protocols to directly compare solid tumor DNA (stDNA) and ctDNA genomic results. The Allegheny Health Network (AHN) Cancer Institute launched an Oncology Biobank and Data Repository initiative in March 2021 for collection of tissue, blood, and comprehensive clinical data to address these issues. Blood was obtained from consenting patients with cancer across the 14 hospitals of the AHN for identification of cancer biomarkers in tumor tissue and blood samples (Oncology Sample Biobank and Data Repository; institutional review board number 2020-258). More than 7000 unique patients contributed to the AHN Biobank by March of 2025, providing initial diagnostic and longitudinal blood samples throughout their course of treatment. All samples in the AHN Cancer Institute biorepository contained comprehensive clinical data using the mCODE framework (Minimal Common Oncology Data Elements) to standardize data collection and ensure efficient data integration and analysis. The aim of this study was to interrogate cell-free DNA of patients with cancer at AHN Cancer Institute using a ctDNA assay in a paired comparison to the clinically validated solid tumor CGP assay. The differences in timing of sample acquisition, tumor stage, interventional therapy, and the presence of other primary tumors were precisely monitored to ensure the highest fidelity comparison attainable. Included in this initiative was the i) standardization of patient blood collection, preservation, and transport of samples to a centralized AHN Genomics Facility, ii) development of a plasma separation and storage protocol to minimize background blood cell–derived germline DNA, iii) implementation of a bead-based, cfDNA purification and quality assurance/quality control protocol to characterize the quantity and integrity of each patient's cfDNA sample, and iv) utilization of a 523-gene NGS panel and analysis pipeline equivalent to the College of American Pathologists/Clinical Laboratory Improvement Amendments–approved CGP solid tumor assay to compare ctDNA results with stDNA profiles from matched FFPE tissue specimens (TruSight Oncology 500: ctTSO500 versus stTSO500; Illumina, San Diego, CA).

## Materials and Methods

### Consent and Sample Collection

This study included male and female patients (aged 18 to 100 years) diagnosed with cancer of any origin who came to AHN for clinical care. Participants signed a Health Insurance Portability and Accountability Act Authorization Statement of informed consent (institutional review board number 2020-258: Oncology Sample Biobank and Data Repository) to contribute de-identified data to a database linking their tumor and blood sequencing data with clinicopathologic tumor information. The protocol included access to tissues from patients with cancer having procedures at 1 of 21 AHN Cancer Institute treatment sites and provision of blood samples obtained during routine, clinical laboratory draws or i.v. therapy at an AHN hospital. Whole blood samples were collected in three, 10-mL Streck, Cell-Free DNA BCT tubes (Streck, LaVista, NE) followed by gentle inversion of each tube 10 times and maintenance of samples at room temperature (18°C to 25°C), according to the manufacturer's recommendation. Streck tubes were encased in bubble wrap and transferred daily by medical courier to the AHN Genomics Facility for processing. Samples subjected to undue agitation, including pneumatic tube system transport, were not included on the basis of previous studies and National Cancer Institute guidelines.^13^^,^^14^ Specimens were coded and tracked for location and temperature during courier transport to the AHN Genomics Facility (Pittsburgh, PA) for processing.

### Isolation of Plasma from Streck Tube Samples

A three-step sequential centrifugation protocol was developed to separate plasma devoid of cells and debris from patient blood samples. This was based on the preliminary observation that a substantial number of cancer patient samples contained visible cell pellets after a second centrifugation. Consequently, a third centrifugation step was incorporated to eliminate these cells as a potential source of germline DNA contamination. Whole blood in Streck tubes was gently mixed and placed in a chilled, swinging bucket rotor for centrifugation at 1600 × *g* (Eppendorf 5810R, S-4-104 rotor, 10 minutes, 4°C, no brake; Eppendorf, Hamburg, Germany). All blood products remained on ice or cold blocks (15 mL × 12; Electron Microscopy Sciences, Hatfield, PA) from the first spin throughout the plasma and buffy coat separation. The plasma layer obtained after centrifugation was transferred to a 5-mL screw cap, conical tube (VWR, Inc., Radnor, PA) without disturbing the buffy coat using a 10-mL pipet tip (Rainin Pipet-Lite LTS L-10 mL; Mettler-Toledo, Greifensee, Switzerland). The plasma was centrifuged in a fixed-angle rotor at 10,000 × *g* (Eppendorf 5425R, FA 10 × 5 rotor, 10 minutes, 4°C, soft brake). The plasma layer was then transferred to a 5-mL screw-cap tube using a 10-mL serologic pipet (catalog number 14955234, Drummond pipet-aid XL; ThermoFisher) without disturbing the cell pellet, and a third spin was performed identically to the previous spin. After the third spin, the plasma was transferred to a 7.6-mL FluidX Tricode tube (Azenta, Burlington, MA) for immediate storage (–80°C) in the Biospecimen repository. The buffy coat layer separated in the initial Streck tube was transferred to a 1.9-mL FluidX Tricode tube (Azenta) using a large-bore, 1000-μL pipet tip (RT LTS 1000 μL; Mettler-Toledo) for long-term storage (–80°C).

### Purification of Cell-Free DNA from Plasma

Frozen plasma was thawed at room temperature (60 minutes) and the volume adjusted as needed (1× phosphate-buffered saline, pH 7.4; catalog number 10010-023; ThermoFisher) for purification of cfDNA using the Apostle MiniMax High Efficiency cfDNA Isolation Kit (catalog number A17622-250; Beckman, Indianapolis, IN). The only protocol modification was the primary Proteinase K digestion incubation that was optimized to 1 hour at 60°C. Cell-free DNA was eluted using magnetic beads in 40 to 50 μL of elution buffer per sample, and 2 μL was assayed for concentration by fluorometry (Qubit Flex; ThermoFisher) using the Qubit dsDNA High Sensitivity kit (catalog number Q32854; ThermoFisher). An aliquot was diluted to 200 to 600 pg/μL, and 2 μL was analyzed on a 5200 Fragment Analyzer (Agilent, Santa Clara, CA) using the HS Large Fragment Kit (DNF-464; Agilent). The analysis software (ProSize Revision 5.0.1.6; Agilent) delineated the DNA fragment distribution from 75 to 300 bp and from 75 to 1200 bp containing cfDNA of sizes associated with nucleosome and linker histones and the 1300 to 150,000 bp domain where background germline DNA from lysed cells was detected when present (Agilent Technologies Application Note, publication number 5994-0521EN: Quality Metrics for Nucleic Acids with the Agilent Fragment Analyzer and Femto Pulse Systems, 2019).^15^ The percentages of those regions were multiplied by Qubit Flex quantitation values to obtain the concentration of each of these variable DNA components for each sample.

### Purification of DNA from FFPE specimens

FFPE tumor specimens were obtained from pathology blocks after review of a correlative hematoxylin and eosin slide by a surgical pathologist (P.L.W.). Cores were punched to obtain pure tumor specimens (1-mm by 0.5-cm punch biopsy) when accessible, or 10 to 20 slides containing unstained sections (5 to 10 μm thickness) were cut for macrodissection of tumor regions demarcated by the reviewer. Unstained slides were deparaffinized in xylene (5 minute immersion × 3), dried, aligned in register with the corresponding hematoxylin and eosin slide, and the tumor tissue was removed using a scalpel (Penblade-size 10, 15, or 22; ThermoFisher) after applying 2 to 4 μL of molecular-grade water (ThermoFisher) to the unstained tissue. Tumor tissue was collected in a 2-mL Eppendorf tube and dehydrated in 500 μL xylene. The xylene was removed after centrifugation (Eppendorf Minispin plus, 10,000 × *g* × 2 minutes), and the samples thoroughly mixed in 100% ethanol (Pharmco, Brookfield, CT) by manual pipetting. The previous centrifugation process was repeated, the ethanol supernatant was removed from the pellet by pipetting, and the samples were allowed to dry. Genomic DNA was extracted from the tumor using the FormaPure XL TNA FFPE Extraction kit (Beckman) according to the Beckman FormaPure XL Manual Extraction Protocol (document number 158133.2; Beckman). DNA from the solid tumor samples (stDNA) was assessed for purity (NanoDrop; ThermoFisher), quantified by fluorometry (Qubit Flex; ThermoFisher), and fragment size and integrity were determined by capillary electrophoresis (5200 Fragment Analyzer; Agilent) as described for cfDNA. DNA samples were advanced to fragmentation if they contained ≥40 ng with fragment sizes >90 bp in the 80 to 20,000 bp range (Agilent Technologies Application Note, publication number 5994-0521EN: Quality Metrics for Nucleic Acids with the Agilent Fragment Analyzer and Femto Pulse Systems, 2019).

### Purification of DNA from Buffy Coat Samples

Frozen buffy coat was thawed at room temperature (40 minutes) before processing, and the volume was adjusted as needed (1× phosphate-buffered saline, pH 7.4; catalog number 10010023; ThermoFisher) for purification using the column-based Quick-DNA Midiprep Plus Kit (D4075; Zymo Research, Irvine, CA), according to the manufacturer's protocol. The only protocol modification was the primary Proteinase K digestion incubation that was optimized to 2 hours at 60°C. Purified buffy coat DNA was checked for purity and quantity, as described in the preceding paragraph for cell-free and solid tumor DNA. An aliquot of the buffy coat genomic DNA was diluted to 3 ng/μL for analysis by capillary electrophoresis (5200 Fragment Analyzer, HS Genomic DNA Kit: DNF-488; Agilent) and advanced for fragmentation with genomic size distribution routinely exceeding 20 kb.

### Sequencing Library Preparation of Solid Tumor, Buffy Coat, and Cell-Free DNA Samples

DNA from solid tumor and buffy coat was prepared according to the Illumina TruSight Oncology 500 Reference Guide (document number 1000000067621 version 10, July 2022; Illumina). The minimum input requirement of 40 ng DNA was sheared (12°C, 10 seconds duration, 20 bursts, 30% duty factor, 1000 cycles/burst) using the Covaris ME220 (Covaris, Woburn, MA) to obtain 80- to 170-bp fragments. The DNA was subjected to end repair and A-tailing reactions, including addition of adapters and unique molecular identifiers to each fragment followed by an index PCR amplification step (15 cycles: 10 seconds at 98°C, 30 seconds at 60°C, and 30 seconds at 72°C) to obtain a yield of 60 to 120 ng/μL. Genomic targets in each sample were selected using hybrid capture probes linked to streptavidin magnetic beads in an overnight incubation (16 to 18 hours at 57°C). After bead separation and clean up, a second enrichment capture step was performed (2 hours at 57°C). After bead capture and clean up, the targets were amplified (18 cycles: 10 seconds at 98°C, 30 seconds at 60°C, and 30 seconds at 72°C) to obtain a final library for sequencing with a peak fragment size range from 325 to 375 bp at a concentration of approximately 20 ng/μL after enrichment. Eight purified DNA libraries were diluted and combined in a final library pool manually normalized to 1.1 pmol/L according to the NextSeq System Denature and Dilute Libraries Guide (document number 15048776 version 16, July 2020; Illumina). Paired-end sequencing (2 × 100 bp or 2 × 150 bp) was performed on the NextSeq 550Dx following the NextSeq550 Sequencing Systems protocol (document number 15069765 version 07, October 2021; Illumina).

Libraries prepared for sequencing from cfDNA followed the Illumina TruSight Oncology 500 ctDNA Reference Guide (document number 1000000092559 version 00, February 2020; Illumina). Briefly, a minimum of 25 to 30 ng cfDNA contained within the 75- to 300-bp DNA domain was required. Library preparation proceeded without fragmentation, including end repair, A-tailing, unique molecular identifier adapter ligation, and index PCR, yielding a concentration of approximately 80 to 170 ng/μL. Two rounds of target enrichment were performed, followed by PCR, to obtain fragments in the 325- to 375-bp range at a concentration of 20 ng/μL. Twenty-four sample libraries were manually normalized (0.65 nmol/L), and paired-end sequencing (2 × 150 bp) was performed on the NovaSeq 6000 according to the NovaSeq 6000 Sequencing System Guide (document number 1000000019358 version 17, September 2022; Illumina).

### Data Analysis Pipeline

FFPE tumor and buffy coat derived binary base call files were generated by the NextSeq Local Run Manager (TruSight Oncology 500 version 2.2 software; document number 10000000151997 version 01, September 2021, Illumina), including read collapsing and error correction to remove sequencing mistakes, low-quality and duplicate reads, deamination artifacts, and high background noise or low coverage regions (Pepe blacklist; Illumina). Binary base call files were converted to aligned BAM files (Burrows-Wheeler Aligner: HG19 reference), followed by local realignment to insertions or deletions and paired-end stitching using the GEMINI software module. Single-nucleotide variants and insertions or deletions were called by PISCES software, multiple nucleotide variants by SCYLLA, and copy number variants by the CRAFT program. Small variants were filtered at read depth ≥100 reference calls and 1% variant allele frequency as the analytical limit of detection. VCF files were annotated by the Nirvana Annotation Engine using public databases (dbSNP, Genome Aggregation Database, 1000 Genomes, ClinVar, RefSeq, and Ensembl). Tumor mutation burden was generated from small variant files across 1.33 Mb of exonic sequence after removing germline variants using TMBRaider software. Mutation annotation format files were generated from JSON files (maftools module of R/Bioconductor: vcf2maf conversion) to interrogate the OncoKB Oncology Knowledge Database version 4.9 for pathogenicity and clinical actionability of variants for US Food and Drug Administration–approved level 1 and Standard Care Level 2 oncology drugs dating back to 1998.^16^^,^^17^

Cell-free DNA libraries sequenced on the NovaSeq6000 (Illumina) were transferred to and processed in Illumina Connected Analytics using the DRAGEN TruSight Oncology 500 ctDNA Analysis software version 2.1 (document number 200028897 version 01, November 2023). Analysis followed the Local Run Manager processing paradigm converting binary base call files to FASTQ and BAM files incorporating the DRAGEN aligner followed by stretched realignment using GEMINI. High background noise or low coverage regions were removed using a Pepe blacklist specific to the ctDNA assay. Single-nucleotide variants and insertions or deletions were called by PISCES, multiple nucleotide variants by SCYLLA, and copy number variants by the CRAFT module. Clonal hematopoietic variants of indeterminate potential in cfDNA (<1% variant allele frequency) were removed using germline (>30% variant allele frequency) and/or the Genome Aggregation Database of population frequencies (<0.001).^16^, ^17^, ^18^ Tumor mutation burden was defined using TMBRaider software. NIRVANA was used to generate annotations based on the same databases as solid tumors. Small variants underwent post-processing filtering for a read depth cutoff at 500 reference calls and 0.1% variant allele frequency as the analytical limit of detection. Mutation annotation format files were generated from cfDNA JSON files to interrogate the OncoKB Oncology Knowledge Database version 4.9, as described in the preceding paragraph. To establish the uniformity of the Local Run Manager pipeline to the cloud-based DRAGEN pipeline associated with the NovaSeq6000, unaligned FASTQ files from stDNA samples were processed through both pipelines, confirming that results were identical despite use of the BWA by the Local Run Manager versus the DRAGEN aligner (Supplemental Figure S1).

### Statistical Analysis

Correlation analysis between solid tissue and circulating tumor data was performed to determine shared and exclusionary variants. Variants within the combined domains of the Pepe blacklists were removed and missense, frameshift, stop-gained, splice region, splice donor, and in-frame insertions and deletions were processed in R using an inner join merging function based on the chromosome start position, end position, reference allele, altered allele, and lower limit of detection to identify mutations in common. A subsequent anti-join function was used to identify mutations unique to the solid tissue, buffy coat, or circulating tumor DNA. Concordance for each of these comparisons was calculated by dividing the total number of variants shared between the plasma and tumor tissue assays by the total number of variants specifically detected in the solid tumors.

The normality of data distribution was assessed using the Kolmogorov-Smirnov test. Between-group differences for normally distributed data were analyzed by *t*-test and analysis of variance, whereas nonnormally distributed data were analyzed by the *U*-test and Kruskal-Wallis test. Linear relationships between data sets were examined using the Spearman rank correlation analysis, and the association between continuous variables was examined using simple linear regression. Continuous data were natural log transformed to normalize skewed distributions and stabilize the variance. Additionally, test characteristics, including sensitivity, specificity, and positive and negative predictive values, were computed for cell-free DNA to predict concordance with therapeutically actionable mutations present in solid tumors.^19^ Statistical analysis was performed using SAS version 9.4 (SAS Institute Inc., Cary, NC) , and *P* < 0.05 was considered statistically significant.

Survival analysis was performed using Cox proportional hazards regression after calculating the time interval for each patient from the date of the initial blood draw to the date of death or censoring on the date of study data lockout (August 9, 2024). For multivariable analysis, patient age was calculated for the date of the initial blood draw; primary tumor site was verified by individual chart review, and tumor stage was assigned according to the American Joint Cancer Committee T/N/M (tumor/node/metastasis) framework. Statistical analysis was performed using SAS version 9.4 or STATA version 16 (StataCorp, College Station, TX) with *P* < 0.05 considered statistically significant.

## Results

**Figure 1:**
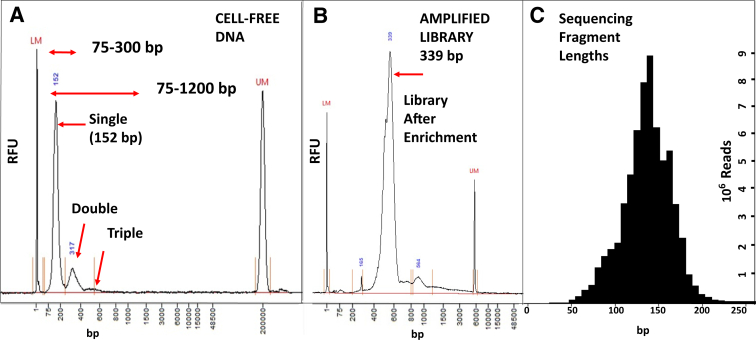
Cell-free DNA (cfDNA), sequencing library, and read length size profiles. **A:** Electrophoretic profile (5200 Fragment Analyzer; Agilent., Santa Clara, CA) showing the distribution of fragment sizes identified in cfDNA from the plasma of a representative patient with lung cancer using our plasma processing and cfDNA purification protocol (see Materials and Methods). The cfDNA samples did not undergo any fragmentation procedure during preparation. Plasma samples included three peaks with diminishing contributions to the overall genomic profile. Base pair (bp) sizes are indicated on the horizontal axis. Lower size marker (LM) = 1 bp; upper size marker (UM) = 200,000 bp. Single: DNA fragments consistent with one nucleosome wrap (152 bp) captured in the 75- to 300-bp range; double: DNA fragments comprising two wraps of a nucleosome (317 bp); triple: DNA fragments of three wraps around a nucleosome. These latter fragments require measurement from the 75- to 1200-bp range. **B:** DNA sequencing library profile for the lung cancer specimen in **A** after addition of sequencing adapters, primer binding sites, flow cell adapter binding sites, and unique molecular identifiers that increased native fragment lengths to a 339-bp peak. This library subsequently underwent hybridization selection for the 523 genes specific to the Allegheny Health Network comprehensive genomic profiling panel. UM = 6000 bp. The prominent peak derives from the single mononucleosomal fragment obtained in **A** with a minor contribution from the double (564) and triple fragment wraps. **C:** The sequencing read length profile was obtained from the FASTQ files using the browser extensible data format for the DNA sequencing library in **B** after enrichment for the 523 genes in the molecular panel. After trimming adapters, primer binding sites, and unique molecular identifiers, the read lengths reflected a single fragment size distribution consistent with the dominant mononucleosomal wrap identified in the original sample. The total number of reads obtained for each read length bin is plotted in millions on the right vertical axis. RFU, relative fluorescence unit.

From inception of the AHN Oncology Biobank initiative in March of 2021 to March 1, 2025, 26,358 diagnostic (initial) and longitudinal samples were processed from 7155 unique patients in the ongoing program. Patients comprised 28 different general tumor types, with largest representation from lung (12.3%), breast (11.9%), colon (10.1%), endometrial (9.7%), and pancreatic (5.9%) cancer specimens (Supplemental Figure S2). Eighty percent of blood specimens were converted to plasma within 29 hours and 96.1% by 72 hours after blood draw (Supplemental Figure S3). cfDNA was processed from the first 874 diagnostic plasma samples spanning these cancer types to determine DNA concentrations, yields, and integrity (fragment sizes) for the collection and processing pipeline. Using a three-spin plasma purification protocol, background germline DNA was rarely seen from blood cell lysis based on high-resolution microcapillary electrophoresis (Figure 1A). Consequently, cfDNA concentrations were evaluated from the samples over the size range from 75 to 1200 bp as a benchmark that captured all visible cfDNA peaks. These peaks were consistent in size with single, double, and triple wraps of DNA fragments protected from degradation through binding to nucleosomes of the histone complex, as originally described by Noll^20^ in 1974. Initial cfDNA purification results revealed a dominant fragment size of 166 bp [mean ($\bar{\text{x}}$) ± SD = 166 ± 5 bp, *n* = 874] consistent with a single nucleosome DNA wrap (Figure 1A). This unimodal distribution was maintained throughout the library preparation and enrichment process (Figure 1B), generating sequencing reads with a mean insert size of 162 bp ($\bar{\text{x}}$ ± SD = 162 ± 25 bp, *n* = 430) (Figure 1C). These data indicated that the DNA undergoing sequencing derived predominantly from cfDNA within the 75- to 300-bp range. Therefore, the requisite 25-to 30-ng loading amount was calculated for the ctDNA sequencing assay using that size range for the measurements.

**Figure 2:**
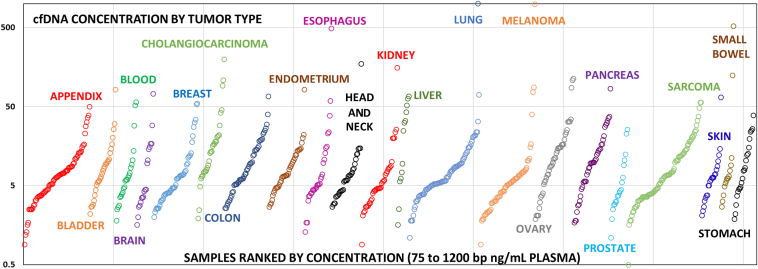
Cell-free DNA (cfDNA) concentrations across tumor types. Concentration in nanograms per milliliter of plasma (ng/mL) for each patient in the study is plotted (log base 10) on the vertical axis for 874 tumors comprising 21 different tumor classifications. Specimens are color coded by tumor type, and individual samples within a tumor type are plotted arbitrarily from low to high concentration (see Materials and Methods and Figure 1A). Only tumor types with a minimum of 10 samples are plotted from among the 874 cfDNA specimens purified for this study. The lowest value collected was 0.50 ng/mL from a patient with osteosarcoma, and highest concentrations were 985 ng/mL from a patient with melanoma and 1132.9 ng/mL from a patient with lung cancer, which exceeds the axis maximum.

The cfDNA concentration of plasma samples (75 to 1200 bp) spanned two to three orders of magnitude across tumor types with a minimum value of 0.50 ng/mL of plasma from a patients with osteosarcoma to a maximum of 1132.9 ng/mL from a patient with lung cancer (Figure 2). These cfDNA concentrations were statistically evaluated for a normal distribution across all 874 samples and 28 tumor types, revealing a probability distribution (Kolmogorov-Smirnov: *P* ≤ 0.01) that led to rejection of the hypothesis of normality due to extremes in concentration values. From the 874 cfDNA patient samples initially evaluated, 373 (42.7%) met the minimum DNA requirement (25 ng) for sequencing from a single Streck tube, two tubes were necessary for 254 (29.1%) of the patients, and 187 (21.4%) required the third tube of blood to reach the minimum yield required for sequencing. Only 60 of 874 samples (6.9%) failed the 25-ng minimum concentration requirement for ctDNA sequencing typically due to inadequate blood draw volume leading to insufficient overall DNA yield. Samples were concentrated under vacuum when necessary to achieve a starting substrate amount within volume requirements for the sequencing assay (Eppendorf Vacufuge Plus). Every plasma sample meeting the starting material requirement according to this protocol revealed cancer coding mutations, including diagnostic and oncogenic mutations common to both solid tumor and cfDNA specimens.

**Figure 3:**
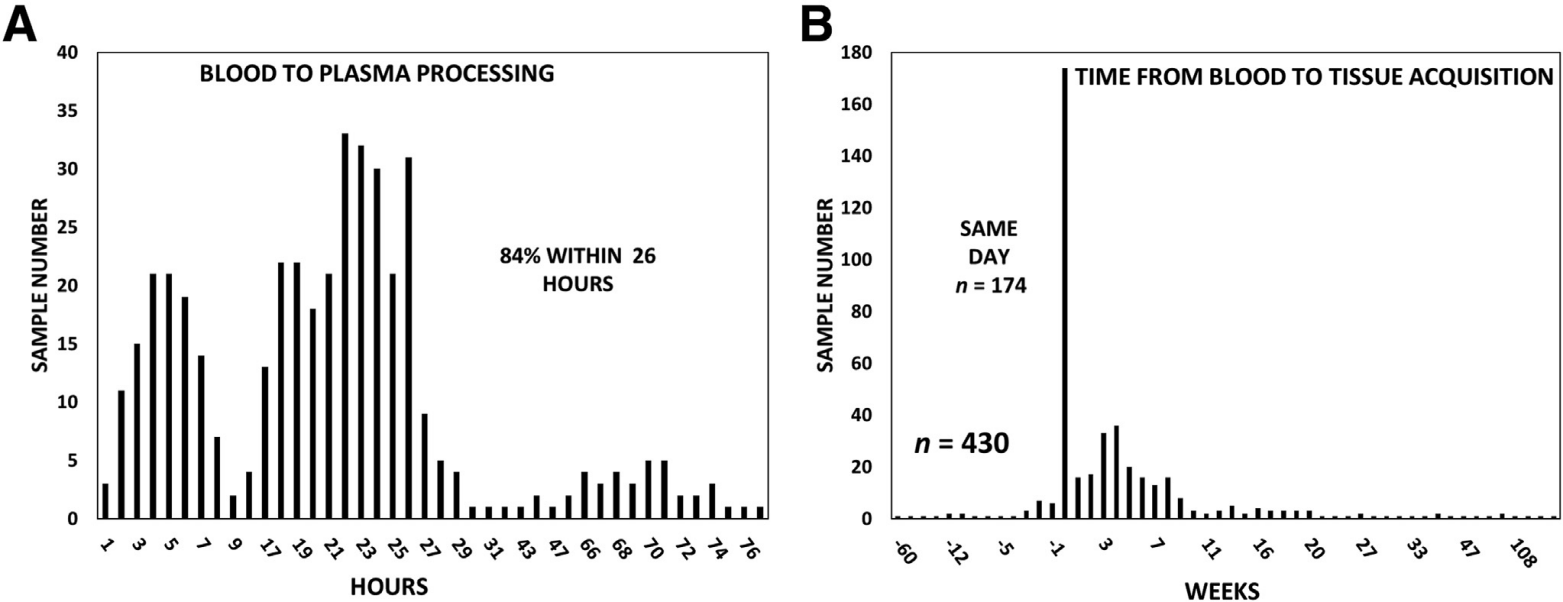
Blood to plasma processing intervals and time proximity of matched blood and tissue acquisition. **A:** Vertical bars indicate the number of blood samples processed to plasma at each hourly time point for the patients in the concordance study, with 0 representing the time of the blood draw. Eighty-four percent of blood samples were processed to plasma within 26 hours of the patient blood draw, and 98% were completed by 72 hours for the patients in this study. **B:** Vertical bars indicate the number of blood samples collected at each time point compared with matching surgical specimen acquisition in the tumor-blood concordance studies. Negative values on the abscissa indicate blood samples obtained before acquisition of tumor tissue, whereas positive numbers indicate samples collected after the tissue was acquired. The highest bar reflects the large number of blood samples that were acquired on the same day before the biopsy or surgical procedure antecedent to anesthesia and surgery protocols. *n* = 430 (**A**).

FFPE blocks were selected as primary diagnostic tumor specimens based on their histopathology and clinical record for comparison to each patient diagnostic blood sample acquired through the biobanking protocol. Residual, matching tumor tissue specimens were obtained for 447 of the 874 patients. These 447 plasma samples were targeted for the concordance sequencing study of matched tumor specimens with a concentration range from 27.6 ng/mL (breast) up to 491.4 ng/mL of plasma (esophagus) measured over the 75- to 1200-bp range that served as the benchmark. Clinical status, histologic grade, tumor stage, and timing of tumor tissue and blood collection were curated for each sample, including delineation of clinical therapeutic interventions before or between collection of blood and tumor specimens (eg, surgery, chemotherapy, immunotherapy, or radiation therapy) (Table 1). Each patient's records were reviewed for previous and/or multiple primary cancers at the time of their initial tumor and blood collection. This approach was undertaken to evaluate challenging preanalytical clinical variables beyond the technical considerations used to standardize collection, transfer, and processing of blood samples with all laboratory procedures performed in the AHN Genomics Facility using uniform assays, instruments, and personnel. Of the 447 patients from whom matching tumor and blood specimens were obtained for sequencing, 17 were subsequently disqualified after their tumor diagnosis was reassigned to a benign status, revised to a noncancer disease status, had multiple active primary tumors, or the FFPE block contained insufficient tissue for the primary tumor to generate sequencing libraries. A total of 361 of the 430 patients who qualified for concordance analysis had matching blood samples processed to plasma and frozen within 26 hours of collection (84%) (Figure 3A) with most blood and tumor samples acquired without prior or intervening therapy (60%) (Figure 3B and Table 1).

**Table 1:** Demographic Characteristics of Patients in the Concordance Study

Age, years	Sex		Cancer status		Plasma processing, hours		Blood vs tumor sample acquisition		Therapy intervention	
63.8 ± 13.3	Male	Female	Stage 1	65	≤24	308	Prior: >20 weeks	1	None	291
	191	239	Stage 2	54	25–48	77	Prior: 5–20 weeks	10	Prior	92
			Stage 3	128	49–72	28	Prior: 1–4 weeks	17	Between	47
			Stage 4	174	73–118	8	Same day	174		
			NA	9			After: 1–4 weeks	102		
			Multiple	45			After: 5–20 weeks	102		
							After: <20 weeks	24		

Age indicates the average for 430 patients at the date of their diagnostic blood draw. Cancer status: American Joint Committee on Cancer clinical stage; Multiple includes patients previously diagnosed and treated for a primary cancer or who had an additional primary cancer diagnosed within the period of the study. Plasma processing indicates the number of patients classified according to the time between their blood draw and storage of their plasma in the –80°C freezer biorepository. Blood vs tumor sample acquisition delineates numbers of patients based on the time differential between acquisition of their blood and tumor samples. Therapy intervention refers to cases where patients underwent therapy (chemotherapy, immunotherapy, or radiotherapy) before acquisition of their samples or therapy was initiated between acquisition of samples.

NA, not available.

The characteristics of the 430 patients in the concordance study were representative of the overall population in the biorepository, with 70% from patients with late-stage cancer, including 45 with a history of multiple cancers (Table 1). The ctDNA variant frequencies and concordance values obtained after sequencing did not conform to a normal distribution (Kolmogorov-Smirnov: *P* ≤ 0.01) and were natural log transformed for analysis of individual variables (Supplemental Figure S4). A high concordance was observed between solid tumor and ctDNA assays for single-nucleotide variant, multiple nucleotide variant, and insertion or deletion mutations. On average, 97% of all solid tumor DNA variants were detected in ctDNA [median (Med) = 98%], including 95% of nonsynonymous, coding variants (Med = 97%) (Table 2). Spearman correlation analysis revealed a highly significant statistical correlation between variants detected in the paired solid tumor and circulating tumor DNA data sets (all variants: *r* = 0.89, *P* < 0.001; coding: *r* = 0.83, *P* < 0.001).

**Table 2:** Concordance of Variants Detected by CGP of Matched Tumors and Plasma Samples

VAL	ALL VAR				CODING VAR				OncoKB VAR			
	BOTH	CF	ST	CONCORD, %	BOTH	CF	ST	CONCORD, %	BOTH	CF	ST	CONCORD, %
AVE	1134.7	39.0	38.6	96.9	165.5	16.0	10.6	94.7	7.5	4.4	2.1	81.8
MED	1123.0	31.0	20.0	98.3	163.0	14.0	5.0	96.9	7.0	4.0	1.0	87.5
MIN	775.0	8.0	7.0		105.0	1.0	0.0		2.0	0.0	0.0	
MAX	1455.0	406.0	531.0		262.0	123.0	176.0		27.0	29.0	32.0	
SD	79.7	43.6	70.7		16.2	12.2	18.0		2.9	3.6	3.1	

									LEVEL 1 THERAPEUTIC (*n* = 430)			
									BOTH	CF	ST	NONE
									233	48	23	126

ALL VAR indicates the overall number of variants, including synonymous and nonsynonymous variants detected by CGP profiling. CODING VAR indicates nonsynonymous, coding variants that alter the RNA transcript, amino acid sequence, and/or peptide sequence if expressed and translated. OncoKB VAR are variants classified using the OncoKB database to determine nonpolymorphic, likely oncogenic, and oncogenic variants, including actionable mutations (level 1) with an approved therapy targeting that mutation. VAL indicates the category of values provided for that column, including AVE for average, MED for median, MIN for minimum, MAX for maximum, and SD for standard deviation for the concordance values. CONCORD indicates the concordance of values detected in the solid tumor tissue that were also identified in the cell-free DNA (cfDNA). BOTH indicates that a specific variant was concomitantly detected in the solid tumor and cfDNA assays for a patient. CF indicates that the variant was detected in the cell-free DNA only. ST indicates that the variant was detected in the solid tumor tissue only. LEVEL 1 THERAPEUTIC refers to the total number of patients with an actionable mutation and their detection by the solid tumor and/or plasma CGP assays.

CGP, comprehensive genomic profiling.

Variants were compared with the OncoKB clinical database version 4.9 (810 genes) to identify oncogenic mutations, including therapeutically actionable mutations from among the 53 level 1 genes in the database (Table 2).^16^^,^^17^ The limited numbers of actionable targets with level 1 therapies in the OncoKB database reduced the scale of these values by an order of magnitude markedly increasing the relative impact of a missing ctDNA variant on concordance. Concurrent stDNA and ctDNA assays revealed potentially oncogenic mutations defined by OncoKB in every tumor and plasma sample tested with actionable level 1 mutations identified in 233 of the 430 patients (54%) by both assays (Table 2). An additional 126 patients with oncogenic mutations did not have pharmacologic therapies associated with their mutation profiles by either assay (29%) (Table 2). Forty-eight of the 430 patients were identified with actionable mutations by only their ctDNA assay. At the same time, 23 patients had actionable mutations found in the stDNA assays alone. Spearman correlation analysis revealed a significant relationship for oncogenic mutations detected in stDNA and ctDNA data sets compared with the OncoKB clinical database (OncoKB: *r* = 0.67, *P* < 0.001) with a concordance of 94.7% in determining therapeutic actionability of at least one variant across each of the 430 patients. These results provided a clinical sensitivity for the ctDNA assay of 91.0% with a specificity, accuracy, and positive predictive value of 100% to detect individual patients with solid tumor mutations assuming the unlikely possibility that the exclusive actionable ctDNA mutations in 48 patients were false positives. Including those ctDNA mutations as true positives increased the overall sensitivity to detect therapeutic actionability to 92.4%.

**Figure 4:**
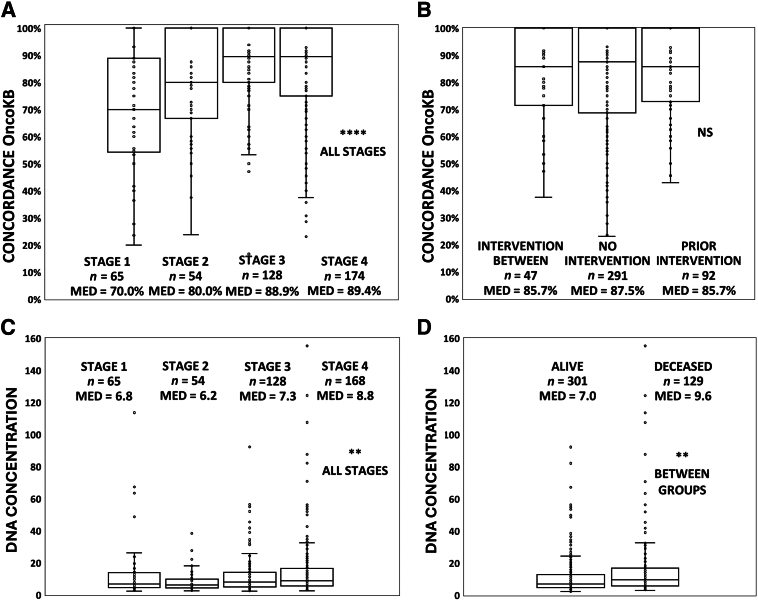
Effect of tumor stage, therapeutic intervention, and vital status on concordance and cell-free DNA (cfDNA) concentration. **A:** Tumors were classified by American Joint Committee on Cancer stage (abscissa: stages 1 to 4) and concordance values defined by OncoKB clinical variant classification plotted on the vertical axis. Concordance demonstrated a statistically significant relationship to stage based on the Kruskal-Wallace test (χ^2^) for a nonparametric data distribution. Data are plotted as box (first and third quartiles) and whisker (horizontal bars = minimum values with maximum limited at 100%) plots, including outliers. The **horizontal line** within each box is the median (MED) with group values reported in the chart. **B:** The group Intervention Between comprises patients who underwent chemotherapy, immunotherapy, or radiation therapy between the collection of their blood and tumor samples. Prior Intervention classification indicates paired blood and tumor specimens from patients who underwent therapeutic interventions in a 60-days window before collection of both samples. The No Intervention group comprises cases where blood and tumor samples were collected before initiation of any clinical treatment. Kruskal-Wallace test (χ^2^: *P* < 0.895) was used; box-and-whisker plots are defined in **A**. **C:** Cell-free DNA concentrations from diagnostic samples were tested for an effect of tumor stage. DNA concentrations are plotted on the vertical axis in ng/mL for the 430 patients in the study. The Kruskal-Wallace test for nonparametrically distributed data revealed a significant effect of tumor stage associated with cfDNA concentration (χ^2^) (see Results). Box-and-whisker plots defined as in **A**. **D:** Patients in the study were classified for vital status on study completion and compared with initial cfDNA concentration values. There was a statistically significant, higher concentration of cfDNA for decedents compared with patients still living within the concordance study (least squares means analysis). Box-and-whisker plots as defined in **A**. ∗∗*P* < 0.01, ∗∗∗∗*P* < 0.0001. NS, not significant.

The number of total, coding, and clinically oncogenic variants in ctDNA and their concordance values in solid tumors were tested for an effect of independent clinical variables, including cancer stage, therapeutic interventions associated with the timing of sample collection, and tumor type. Although concordance was high across these samples regardless of stage for overall and coding variants, there was a significant difference detected for OncoKB clinical variants, indicating that concordance increased progressively from stage 1 and 2 to higher values obtained for stage 3 and 4 patients (Kruskal-Wallace test; χ^2^: *P* < 0.0001) (Figure 4A). These differences were largely driven by breast, lung, endometrial, ovarian, pancreatic, and sarcoma cancer patient specimens that comprised both early- and late-stage patients in contrast to appendiceal, cholangiocarcinoma, colorectal, esophageal, and renal cancers that incorporated predominantly stage III and IV cases. This was particularly striking in patients with ovarian and sarcoma cancer, where median concordance values for stage III and IV within the OncoKB database reached 100% for each of these tumor types (Table 3). There was no significant effect of interventional status or tumor type on concordance values of overall, coding, or clinical variants classified by OncoKB (Figure 4B and Table 3).

**Table 3:** Variants Detected by CGP in Matched Tumor and Plasma Samples According to Tumor Type

TUMOR	CODING VAR				OncoKB VAR				LEVEL 1 THERAPEUTIC			
	BOTH	CF	ST	CONCORD, %	BOTH	CF	ST	CONCORD, %	BOTH	CF	ST	NONE
Appendix												
AVE	163.5	10.5	7.5	95.9	6.8	3.0	1.8	81.3	22	3	3	17
MED	159.0	9.0	5.0	96.9	7.0	3.0	1.0	85.7				*n* = 45
SD	14.8	5.9	12.7		1.9	2.5	1.9					
Bladder												
AVE	161.2	18.8	11.8	93.3	7.9	5.3	3.0	71.4	9	1	0	2
MED	159.5	15.5	10.0	94.3	6.5	4.0	3.0	71.4				*n* = 12
SD	9.7	17.2	7.9		3.2	5.9	1.9					
Blood												
AVE	168.4	23.1	11.6	94	8.3	6.1	1.1	89.9	4	1	0	6
MED	168.0	19.0	6.0	97	8.0	5.0	1.0	90.9				*n* = 11
SD	17.0	9.7	12.6	6	2.9	4.0	1.3					
Breast												
AVE	163.2	12.7	11.5	94.2	7.2	4.0	2.0	79.4	24	2	3	7
MED	160.5	9.5	5.5	96.9	7.0	3.0	1.0	82.3				*n* = 36
SD	15.5	9.3	17.7		2.5	4.8	1.8					
CHOLANG												
AVE	165.9	15.6	5.8	96.7	7.1	3.7	1.5	84.0	9	2	0	5
MED	161.5	14.0	4.0	97.9	7.0	4.0	1.0	87.5				*n* = 16
SD	14.6	10.8	5.4		2.1	2.0	2.4					
Colon												
AVE	171.0	17.2	17.4	92.0	9.0	4.6	3.3	78.0	24	1	4	8
MED	171.0	15.0	6.0	96.5	8.0	4.0	1.0	85.7				*n* = 37
SD	18.0	19.4	26.5		4.2	4.3	4.2					
ENDOMET												
AVE	170.6	13.6	10.1	94.8	6.9	3.9	3.5	72.1	18	2	3	9
MED	167.0	13.0	7.0	95.7	7.0	3.5	2.0	73.2				*n* = 32
SD	18.6	6.6	12.0		2.2	2.8	4.0					
ESOPHAG												
AVE	162.0	17.4	13.9	93.0	8.0	5.3	1.9	84.1	3	1	0	7
MED	165.0	18.0	8.0	95.2	9.0	5.0	1.0	90.0				*n* = 11
SD	8.1	8.6	21.1		1.8	3.5	2.6					
H & N												
AVE	165.7	22.5	15.3	93.0	7.5	4.0	2.1	83.9	9	1	1	0
MED	165.0	17.0	5.0	96.8	8.0	3.0	1.0	88.9				*n* = 11
SD	11.0	22.8	25.1		2.1	3.3	3.6					
Kidney												
AVE	163.7	16.3	7.8	95.5	8.1	5.6	1.7	82.3	9	1	0	4
MED	160.5	16.0	8.5	95.2	9.0	5.5	2.0	80.6				*n* = 14
SD	14.1	6.5	4.1		2.1	2.9	1.4					
Lung												
AVE	173.3	20.4	12.8	93.5	8.3	5.1	2.2	79.0	25	6	3	7
MED	172.0	17.0	7.0	95.8	8.0	4.0	2.0	83.3				*n* = 41
SD	15.8	11.3	15.0		2.9	2.9	2.0					
Ovary												
AVE	161.2	19.1	4.3	97.4	7.8	5.6	0.8	91.8	16	7	1	6
MED	163.0	16.0	3.0	98.1	7.5	5.0	0.0	100.0				*n* = 30
SD	11.4	12.4	3.3		2.2	5.0	1.1					
Pancreas												
AVE	159.5	12.5	6.5	96.4	7.2	4.3	1.4	84.1	10	3	1	5
MED	161.0	13.0	4.0	97.4	7.0	4.0	1.0	88.9				*n* = 19
SD	10.4	6.4	10.5		2.2	3.1	1.5					
Sarcoma												
AVE	162.0	16.5	9.3	95.5	6.5	4.3	1.1	89.6	20	7	1	14
MED	163.5	15.5	3.5	97.8	6.0	3.0	0.0	100.0				*n* = 42
SD	16	11	18		2	3	2					
Stomach												
AVE	161.3	17.7	31.1	87.7	6.6	6.2	6.4	64.5	4	3	3	1
MED	165.0	17.0	7.0	96.0	7.0	6.0	2.0	71.4				*n* = 11
SD	8.6	7.6	48.8		2.0	3.0	9.1					

All labels are displayed according to Table 2; *n* = the number of patients in each tumor cohort. The LEVEL 1 THERAPEUTIC classification indicates the number of individual patients with an actionable pharmacotherapy who were identified by both assays (BOTH) or independently by the cell-free assay alone (CF) or found only in the solid tumor tissue (ST).

CGP, comprehensive genomic profiling; CHOLANG, cholangiocarcinoma; ENDOMET, endometrial cancer; ESOPHAG, esophageal cancer; H & N, head and neck cancer.

The relationship of preanalytical clinical variables was evaluated on the cfDNA plasma concentrations of diagnostic blood samples (Figure 4, C and D). The cfDNA concentration values for the 430 patients in the concordance study were nonparametrically distributed (Kolmogorov-Smirnov: *P* ≤ 0.01), and the Kruskal-Wallace test indicated a significant overall effect of tumor type on cfDNA concentration values (χ^2^: *P* ≤ 0.0001). Iterative paired comparisons were performed between every tumor type combination to determine which tumor types were responsible for the significant differences (Dwass-Steel-Critchlow-Fligner method for pairwise comparisons). Only the cholangiocarcinoma cohort concentration values ($\bar{\text{x}}$ = 26.4 ng/mL of plasma, Med = 13.6 ng/mL of plasma) were determined to have a statistically significant difference among all tumor combinations tested after false discovery correction for multiple testing. These significant differences related to cholangiocarcinoma were specific to lower concentration values detected for appendiceal ($\bar{\text{x}}$ = 8.8 ng/mL, Med = 6.4 ng/mL; *P* ≤ 0.01), breast ($\bar{\text{x}}$ = 9.7 ng/mL, Med = 5.1 ng/mL; *P* ≤ 0.03), and kidney samples ($\bar{\text{x}}$ = 10.7 ng/mL, Med = 4.7 ng/mL; *P* ≤ 0.02) compared with the cholangiocarcinoma cohort.

There was an effect of cancer stage on overall cfDNA concentration values based on the Kruskal-Wallace test for nonparametric data (χ^2^: *P* ≤ 0.0015) (Figure 4C). Pairwise multiple comparisons across all combinations of stages (Dwass-Steel-Critchlow-Fligner test) determined differences to be specific to stage II versus either stage III (*P* < 0.05) or stage IV values (*P* < 0.0006), spanning the lowest and highest median concentration values (Figure 4C). Also, cfDNA values were compared from naïve samples, where the patient had no treatment versus blood samples matched to tissue where patients underwent chemotherapy, immunotherapy, or radiation therapy within 60 days before blood draw or blood drawn after surgical resection within the 60-day time frame. There was no statistical difference in the values obtained between these two groups (no treatment *n* = 208: $\bar{\text{x}}$ = 12.6 ng/mL, Med = 7.7 ng/mL; treatment *n* = 222: $\bar{\text{x}}$ = 15.9 ng/mL, Med = 7.6 ng/mL).

The relationship of initial cell-free DNA plasma concentrations was compared with vital status (alive versus deceased) of the patients, many of whom were still undergoing care. This analysis controlled for incremental but significant differences in cfDNA concentrations based on stage, tumor type, and the age of living patients who were significantly younger than decedents (alive *n* = 301: $\bar{\text{x}}$ = 55.8 ± 10.4 years; deceased *n* = 129: $\bar{\text{x}}$ = 67.7 ± 12.4 years; *P* < 0.001). A highly significant difference was detected on the basis of viability of the 430 patients tested for concordance using least squares means analysis (alive: $\bar{\text{x}}$ cfDNA = 11.1 ng/mL of plasma, Med cfDNA = 7.0 ng/mL of plasma; deceased: $\bar{\text{x}}$ cfDNA = 21.5 ng/mL of plasma, Med cfDNA = 9.6 ng/mL of plasma; *P* < 0.002) (Figure 4D). On the basis of this finding in the matched 430 specimen cohort, the analysis was expanded to all 874 patients from whom cfDNA was purified and for whom vital status was available. Again, living patients were significantly younger than decedents at their initial blood draw (alive: $\bar{\text{x}}$ = 62.0 ± 13.4 years; deceased: $\bar{\text{x}}$ = 67.8 ± 12.4 years; *P* < 0.001). However, cfDNA plasma concentration values were significantly elevated in patients who were deceased versus living (end of March 2024), controlled for the effect of patient age (alive *n* = 665: $\bar{\text{x}}$ cfDNA = 9.1 ng/mL, Med cfDNA = 5.7 ng/mL; deceased *n* = 209: $\bar{\text{x}}$ cfDNA = 34.0 ng/mL, Med cfDNA = 10.1 ng/mL; *P* < 0.0001).

**Figure 5:**
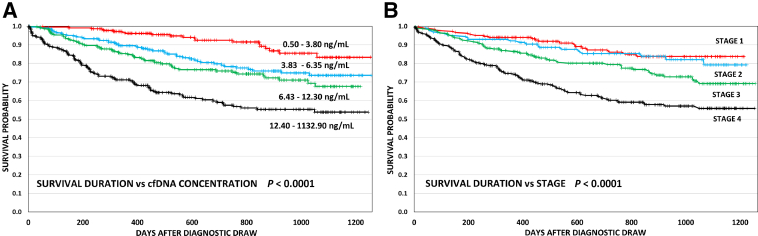
Kaplan-Meier survival probability curve versus cell-free DNA (cfDNA) concentration and tumor stage. **A:** Overall survival curves plotted for 874 patients with cancer in four cohorts based on cfDNA quartile concentrations of initial blood samples. The concentration range for each curve is indicated adjacent to each plotted curve. Statistical analysis was corrected for age differences (see Results). Survival prognosis progressively declined across cohorts based on increasing cfDNA concentration values (χ^2^: *P* < 0.0001). **B:** Survival curves included 826 tumor patients based on American Joint Committee on Cancer stage at primary tumor diagnosis. Statistical significance was derived from log-rank survival analysis. **A** and **B:** See Supplemental Table S1 for hazard ratios generated for the Kaplan-Meier plots. *n* = 665 living (**A**); *n* = 209 decedents (**A**).

On the basis of the vital status analysis, the relationship of cfDNA concentration in our patients to overall duration of survival was tested as a continuous variable from the time of their initial blood draw by a Cox proportional hazards survival regression model, and a significant effect was found after adjusting for age, stage, and tumor type (Figure 5A) (*n* = 874, χ^2^: *P* ≤ 0.0001). The hazard ratio of the cfDNA relationship was 1.005, indicating that probability of death increased by 0.5% per unit increase in cfDNA. These results were stratified by cfDNA concentration quartiles in a Kaplan-Meier survival analysis that indicated a potentially prognostic relationship existed between the initial baseline cell-free DNA concentration and the duration of patient survival (Figure 5A and Supplemental Table S1). Also, the relationship of tumor stage to survival probability was analyzed independent of the initial diagnostic cfDNA concentration in patients for whom comprehensive data were available (*n* = 826) (Figure 5B and Supplemental Table S1). There was a significant effect of diagnostic tumor stage on duration of survival among the patients (χ^2^: *P* ≤ 0.0001). Adjusting for the effect of tumor stage, the hazard ratio for cfDNA decreased to 1.004 (χ^2^: *P* < 0.0001) (Supplemental Table S1).

**Figure 6:**
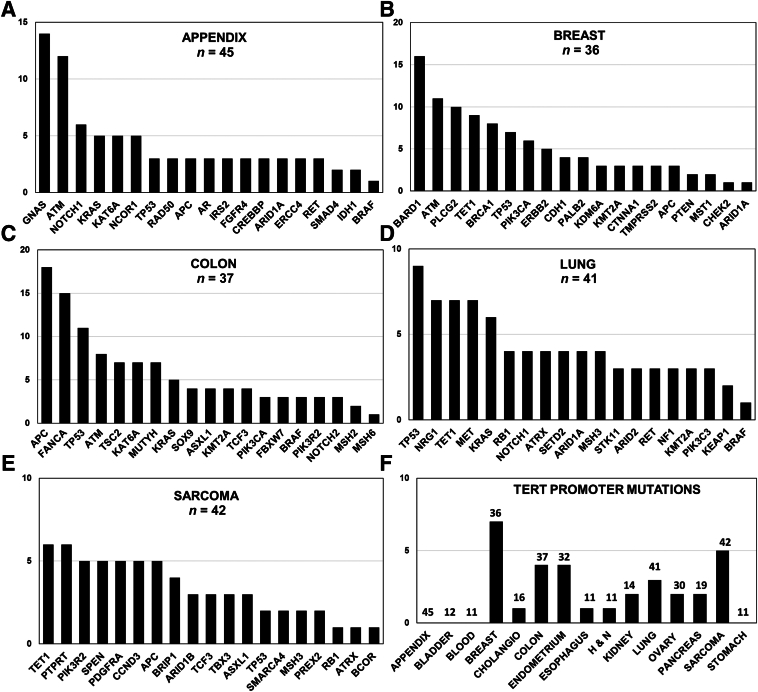
Coding gene mutations detected by solid tumor and plasma assays classified by individual tumor type and telomerase reverse transcriptase (*TERT*) promoter mutations. **A**–**E:** Tumor-specific coding mutations (likely pathogenic or pathogenic) ranked by total number per gene and separated on the basis of individual tumor types: appendiceal (**A**); breast (**B**); colon (**C**); lung (**D**); and sarcoma (**E**). All germline and clonal hematopoiesis of indeterminate potential variants were removed by sequencing and comparative analysis. The vertical axis indicates the number of patients indicated by individual bars containing at least one variant for the gene specified on the abscissa. **F:** Oncogenic *TERT* promoter, tumor-specific mutations detected by solid tumor and plasma assays classified by individual tumor type. The bars indicate the number of patients with oncogenic, noncoding variants in the *TERT* promoter indicated on the ordinate for each tumor type specified on the abscissa. The numbers above each bar indicate the total patients tested for pathogenic *TERT* mutations in each tumor cohort. The absence of a bar indicates that no *TERT* promoter mutations were detected.

Variant profiles were interrogated across tumor types to evaluate their mutational landscapes defined concurrently by stDNA and ctDNA assays. Concordant, tumor-specific mutations were identified after removal of germline and clonal hematopoiesis of indeterminate potential variants and classified against the OncoKB database for genes that play a role in cancer, excluding benign or likely neutral variants.^16^, ^17^, ^18^ The global patient profile of clinical somatic mutations in cancer driver genes detected among the patients comprised 125 established oncogenes and 102 canonical tumor suppressor genes.^21^, ^22^, ^23^ Also, *TERT* promoter variants identified specifically in the OncoKB database as pathogenic, noncoding mutations, indicative of telomerase activation associated with carcinogenesis, were evaluated.^24^ Appendiceal cancers are rare in the United States (one to two/million annually) but were well represented in the patient cohort. *GNAS*, *KRAS*, *TP53*, and *ATM* mutations have been identified as critical drivers of this disease and were primary variants detected in the blood of the patients with appendiceal cancer (Figure 6A).^25^^,^^26^ *PIK3CA*, *TP53*, and *CDH1* mutations were identified in previous liquid biopsy studies of patients with breast cancer and were similarly identified in our patients (Figure 6B).^27^^,^^28^ Also, shared somatic SNPs in *BARD1*, *ATM*, and *BRCA1* genes were detected among the patients with breast cancer at much higher frequencies than predicted by Genome Aggregation Database population allele frequencies (AHN versus Genome Aggregation Database: *BARD1* = 44% versus 1.4%, *ATM* = 31% versus 3.2%, *BRCA1* = 22% versus 4.6%).^27^ Among colon cancer genotypes, *APC* mutations were the most common shared variant along with *TP53*, *KRAS*, and *PIK3CA* mutations known to be oncogenic drivers of colorectal tumors (Figure 6C).^26^^,^^29^ *TP53*, *KRAS*, and *RB1* were among the most prevalent driver mutations in the lung cohort that also included mutations in *NRG*, *TET1*, and *MET* (Figure 6D).^26^^,^^30^ Tumor-specific, epidermal growth factor receptor mutations were infrequently detected within the lung cancer cohort, but 11 patients had a germline missense variant (chromosome 7: 55229255 G>A; p. Arg521Lys) reported in Catalogue of Somatic Mutations in Cancer to be prevalent in patients with lung cancer. Patients with sarcoma were notable for their limited number of shared variants, as previously reported.^31^ Sarcoma variants were identified in *TET1*, *PTPRT*, and *PIK3R2* along with a small number of mutations in important tumor suppressor genes, *TP53* and *RB1* (Figure 6E).^31^ All samples were tested for oncogenic *TERT* promoter mutations that support proliferation of neoplastic cells and serve as an early prognostic indicator.^32^ Thirty-nine patients were affected with breast, sarcoma, colon, endometrium, and lung cancer cohorts containing *TERT* mutations at levels comparable to other recurrent mutations in their tumors (Figure 6F).

## Discussion

A primary goal of this study was to evaluate and standardize all preanalytical variables required for comprehensive genomic profiling of cancer patient blood samples across the Allegheny Health Network. Concordance values obtained in precisely matched tumor and blood specimens after optimization of these protocols reached 97% for coding mutations and 88% for specification of interventional chemotherapy or immunotherapies using blood samples. We selected the CGP assay based on its comprehensive design and performance characterization in previous solid tumor and circulating tumor DNA studies.^33^^,^^34^ Evaluation of the blood collection processes incorporating Streck Cell-Free DNA BCT tubes led us to i) eliminate use of pneumatic tube transfer, ii) exclude sample storage in outdoor lockboxes subjected to varying environmental temperatures, iii) individually wrap tubes in bubble wrap to reduce blood cell lysis, and iv) use a dedicated medical courier service for efficient specimen transport to the AHN central genomics facility. These adjustments combined with three-spin, rapid blood to plasma processing and bead-based cfDNA purification contributed to DNA profiles with minimally detectable background germline DNA. All patient clinical data were obtained under local institutional review board approval with informed consent of each patient allowing access to dynamically changing clinical information in contrast to a static, de-identified patient profile available in institutional review board exempt studies. Consequently, we were able to rapidly identify exceptional conditions that could impact results such as specimen reclassification to benign, from an unknown primary, or of noncancerous disease origin (approximately 7% of initial cases). This also allowed us to obtain precise and updated clinical stage, histologic status, and therapeutic intervention dates for each patient as their diagnosis and treatment plan evolved.

Optimization of blood collection, transfer, and processing protocols allowed us to standardize downstream technical quality assurance/quality control protocols for evaluating cfDNA concentration, yield, and fragment size. High-resolution, microcapillary electrophoresis combined with cfDNA fluorometry was applied uniformly across all samples to quantitate cfDNA nucleosome related fractions and, in the absence of background germline DNA, utilize the 75- to 1200-bp DNA size range as a benchmark for screening cfDNA plasma concentrations across the patient population. This systematic approach assured accurate quantitation of starting substrate, resulting in successful genomic sequencing and informative diagnostic results generated from each cfDNA sample sequenced. These results were notable given that the tumor fraction of ctDNA versus normal DNA was likely changing across the specimens based on tumor type and stage and consistent with the broad range of cfDNA values that were obtained regardless of tumor type and stage. We speculate that the extra precautions taken to rapidly process samples, minimize blood cell lysis, and remove all blood cells during plasma separation contributed to dependable, high-resolution ctDNA sequencing results from each sample tested.

The approach contrasted with liquid biopsy protocols that rely on volumetric standards of plasma input that may yield negative results due to large biologic variability of cfDNA concentrations or differing amounts of blood cell–derived germline DNA. The broad range of cfDNA concentrations across cancer types encountered was consistent with previous publications where methodological details were available for direct comparison to our data.^5^^,^^35^, ^36^, ^37^, ^38^, ^39^ An unexpected finding from the study was that higher concentrations of cfDNA in the diagnostic samples were associated with a reduced duration of survival independent of primary tumor site and stage. This was initially detected in the 430 paired samples of the concordance study, and the statistical significance extended to all 874 patients in the study from whom we collected cfDNA when we expanded our testing. A limited number of previous studies among many have demonstrated a similar correlation, albeit directed at small numbers and within discrete cancer types.^33^^,^^35^, ^36^, ^37^ The results support these previous findings, indicating the importance of tumor type and stage in overall cancer outcomes, but indicate that cfDNA concentrations have independent prognostic value as a biomarker in the assessment of patients with cancer before therapy and in the absence of knowledge regarding tumor fraction, mutation profiles, or overall tumor mutation burden. In fact, we found a prognostic association of cfDNA concentration with overall survival duration that was independent of patient age and sex, primary tumor site, and stage.

Implementing genomic assays in-house proved invaluable in controlling preanalytical variables, establishing internal quality assurance/quality control benchmarks, and reducing turnaround times. The results demonstrated efficacy of the ctDNA CGP assay for identification of clinically relevant oncogenic mutations found in the diversity of tumor types and patients treated across the integrated, community health care network. A significant advantage of the local testing approach used was the unbroken stewardship of all specimens and data within the Allegheny Health Network that comprises a critical resource for designing patient-specific, follow-up assays and represents the framework for a precision medicine initiative within the health care network. If the high fidelity of ctDNA variants in mirroring tumor content in this study extends throughout the patient treatment paradigm, longitudinal blood samples after tumor excision could serve as a biomarker bellwether of the response of residual tumor or metastases to the treatment paradigm. It remains to be determined if diminishing mutation levels or a reduced cfDNA tumor fraction represent challenges that will require higher sensitivity than NGS methods to quantitatively interrogate a patient's response to therapy and alter therapeutic choices or de-escalate treatment protocols. However, these data demonstrated that precise control and quality assurance/quality control of preanalytical variables, including rapid processing of blood samples, can deliver high concordance and acuity of ctDNA testing when performed on-site within a large community health care network.

We used a uniform sequencing and analysis platform across all assays to control for variability in these complex technical processes. All samples contained clinically relevant oncogenic mutations with 60% of the tumors and 65% of the plasma samples containing at least one therapeutically actionable mutation based on the OncoKB database. We identified primary somatic mutations within genes and across individual tumor types that have been identified in other recent studies of ctDNA.^4^^,^^5^^,^^9^^,^^10^ These results provided support for the idea that ctDNA from plasma can serve at the least, as a surrogate biomarker substrate when tumor tissue is not available for diagnosis but may also provide important insight regarding tumor status in longitudinal samples obtained from a patient undergoing treatment. Commercial cfDNA plasma assays using CGP panels have received US Food and Drug Administration approval as a companion diagnostic for treatment of patients with cancer based on the presence of a few specific mutations. The current study incorporated the evaluation of the assay for diagnostic tumor variants across the entire coding domain of 523 genes plus the TERT promoter region, demonstrating high concordance with tumor tissue (Med = 96.9%) and high clinical sensitivity (91%) for therapeutic actionability among the diverse cancers encountered in the health system. Most ctDNA samples (93%) in the study contained additional clinical oncogenic mutations beyond those concurrently detected in the FFPE tumor specimens. This suggests that ctDNA may hold the key to understanding the full spectrum of mutations within a heterogeneous tumor and potentially reveal hidden micrometastases or newly emerging primary cancers. Further studies are required to determine if these ctDNA-specific targets reflect intratumor molecular heterogeneity of the FFPE tissue specimens or if these variants were associated with micrometastatic sites or primary cancers as yet undetected in these patients at the time of their diagnostic blood draw. In either case, these findings specific to cfDNA sequencing represent potentially advantageous information pertinent to treatment of the patient.

## Disclosure Statement

C.J.H. serves in a consultant/advisory role for Astra Zeneca, Biotheranostics, and Gilead; and has been on the speaker's bureau for AstraZeneca and Diiachi Sanyo. A.H.Z. serves in a consultant/advisory role for Previse, Delfi Diagnostics, Prognomiq, BilliontoOne, and Gilead; has received research funding from Eli Lilly, Prognomiq, Delfi Diagnostics, BilliontoOne, Genece Health, Exai Bio, Myriad Genetics, and Tempus; and has equity interest in Previse, TG Therapeutics, and Gritstone Bio. J.N. serves in a consultant role for AstraZeneca and Eisai Inc.; has received research funding from Myriad Genetics; and has been on the speaker's bureau for AstraZeneca, Eisai, and Merck. B.B. and E.D. are full-time employees of Illumina, Inc. C.J.A., D.L.B., R.M.B., O.C., L.B.E., P.H.G., L.G., H.K.K., T.L.L., W.A.L., P.P., P.E.S., S.P., R.S., T.R., K.M.T., and P.L.W. have no commercial relationships to declare.

## Supplemental Data

Supplemental Figure S1 Schematic of substrate processing workflow and timelines. The individual columns delineate major steps and timeframes for processing the plasma, buffy coat cells, and tumor tissue derived from formalin-fixed, paraffin-embedded (FFPE), tumor tissue blocks (see Materials and Methods for details). Significant time delays were often associated with the procurement of the solid tumor tissue specimens, and many required additional recuts to obtain enough to complete testing. Slides were microdissected to obtain tumor cells in a minimal background of normal cells. ctDNA, circulating tumor DNA.

Supplemental Figure S2 Distribution of plasma samples in the Allegheny Health Network Cancer Institute Biorepository. Horizontal bars delineate the numbers of unique patients collected for each cancer type indicated on the vertical axis from March 1, 2021, until March 1, 2025, from a total of 7155 unique patients. Each patient provided an initial, diagnostic whole blood sample (three Streck cell-free DNA BCT Tubes) that was processed to plasma for storage in our –80^0^C freezer biorepository. Longitudinal samples are not included in this histogram.

Supplemental Figure S3 Blood to plasma processing times. Vertical bars indicate the number of blood samples processed to plasma at each hourly time point from inception of the Biobank to March 1, 2025, with the 0 time point representing the time of the blood draw. These data include longitudinal samples obtained from patients that were collected and transported in the same manner as initial diagnostic samples. Eighty percent of blood samples (80.3%) were processed to plasma within 29 hours of the patient blood draw, and 96.1% were completed by 72 hours.

Supplemental Figure S4 Variant detection and distribution in concordance samples. Vertical bars indicate the number of overall variants found in the 430 matched tumor tissue and cell-free DNA samples that underwent sequencing in this study. Individual bins indicate the number of variants detected in samples with each bin increasing in variant number across the range we detected. The circulating tumor DNA variant frequencies and concordance values obtained after sequencing did not conform to a normal distribution (Kolmogorov-Smirnov: *P* ≤ 0.01) and were natural log transformed for analysis of individual variables.
