# Supplementary material for: Cell-Free DNA, Tumor Molecular Concordance, and Clinical Correlates of Patients with Cancer Treated in a Large Community Health Care Network
Source: J Mol Diagn. 2025 Jun 25;27(9):882–98. doi: 10.1016/j.jmoldx.2025.05.007 (PMC12489365; doi:10.1016/j.jmoldx.2025.05.007)
Supplement: Supplemental Figure S1 — Schematic of substrate processing workflow and timelines. The individual columns delineate major steps and timeframes for processing the plasma, buffy coat cells, and tumor tissue derived from formalin-fixed, paraffin-embedded (FFPE), tumor tissue blocks (see Materials and Methods for details). Significant time delays were often associated with the procurement of the solid tumor tissue specimens, and many required additional recuts to obtain enough to complete testing. Slides were microdissected to obtain tumor cells in a minimal background of normal cells. ctDNA, circulating tumor DNA. [file mmc1.pdf]

3 Tissue Types:	Cell free (CF) DNA	Buffy Coat (BC) DNA	FFPET (ST) DNA & RNA
	⇩	⇩	⇩
**Collection, Accession, & Processing Sample**	Blood to Plasma	Cells from Blood	FFPE Tumor Tissue
Time	>80% 24 hours	>80% 24 hours	Varies widely
	⇩	⇩	⇩
**Sample Extraction**	Bead-based Large Volume (DNA)	Column-Large Volume (DNA)	Bead-based TNA (RNA before DNA)
Time	8 hours	8 hours	48 hours
	⇩	⇩	⇩
**Sample Library Preparation**	ctDNA TSO500	ST TSO500	ST TSO500
Time	48 hours	72 hours	96 hours
	⇩	⇩	⇩
**Sample Sequencing**	NovaSeq	NextSeq or Novaseq	NextSeq
Time	~48 hours	24 - 48 hours	~24 hours
	⇩	⇩	⇩
**Primary, Secondary Analysis to VCF**	NovaSeq	NextSeq or Novaseq	NextSeq
Time	~48 hours	24 - 48 hours	< 48 hours
