# Supplementary figures and images for: Cell-Free DNA, Tumor Molecular Concordance, and Clinical Correlates of Patients with Cancer Treated in a Large Community Health Care Network
Source: J Mol Diagn. 2025 Jun 25;27(9):882–98. doi: 10.1016/j.jmoldx.2025.05.007 (PMC12489365; doi:10.1016/j.jmoldx.2025.05.007)

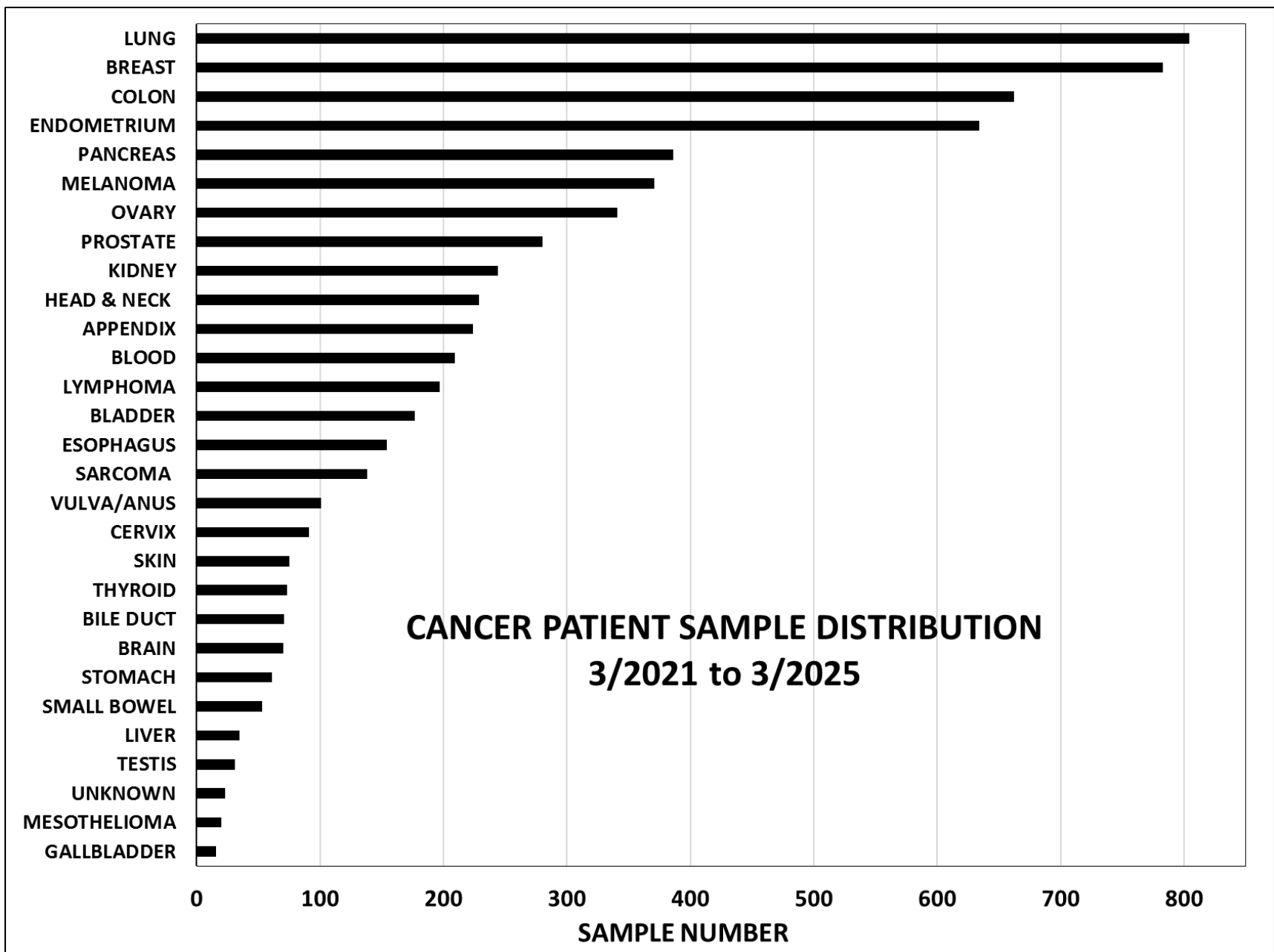

Supplement: Supplemental Figure S2 — Distribution of plasma samples in the Allegheny Health Network Cancer Institute Biorepository. Horizontal bars delineate the numbers of unique patients collected for each cancer type indicated on the vertical axis from March 1, 2021, until March 1, 2025, from a total of 7155 unique patients. Each patient provided an initial, diagnostic whole blood sample (three Streck cell-free DNA BCT Tubes) that was processed to plasma for storage in our –800C freezer biorepository. Longitudinal samples are not included in this histogram. [file mmc2.pdf]

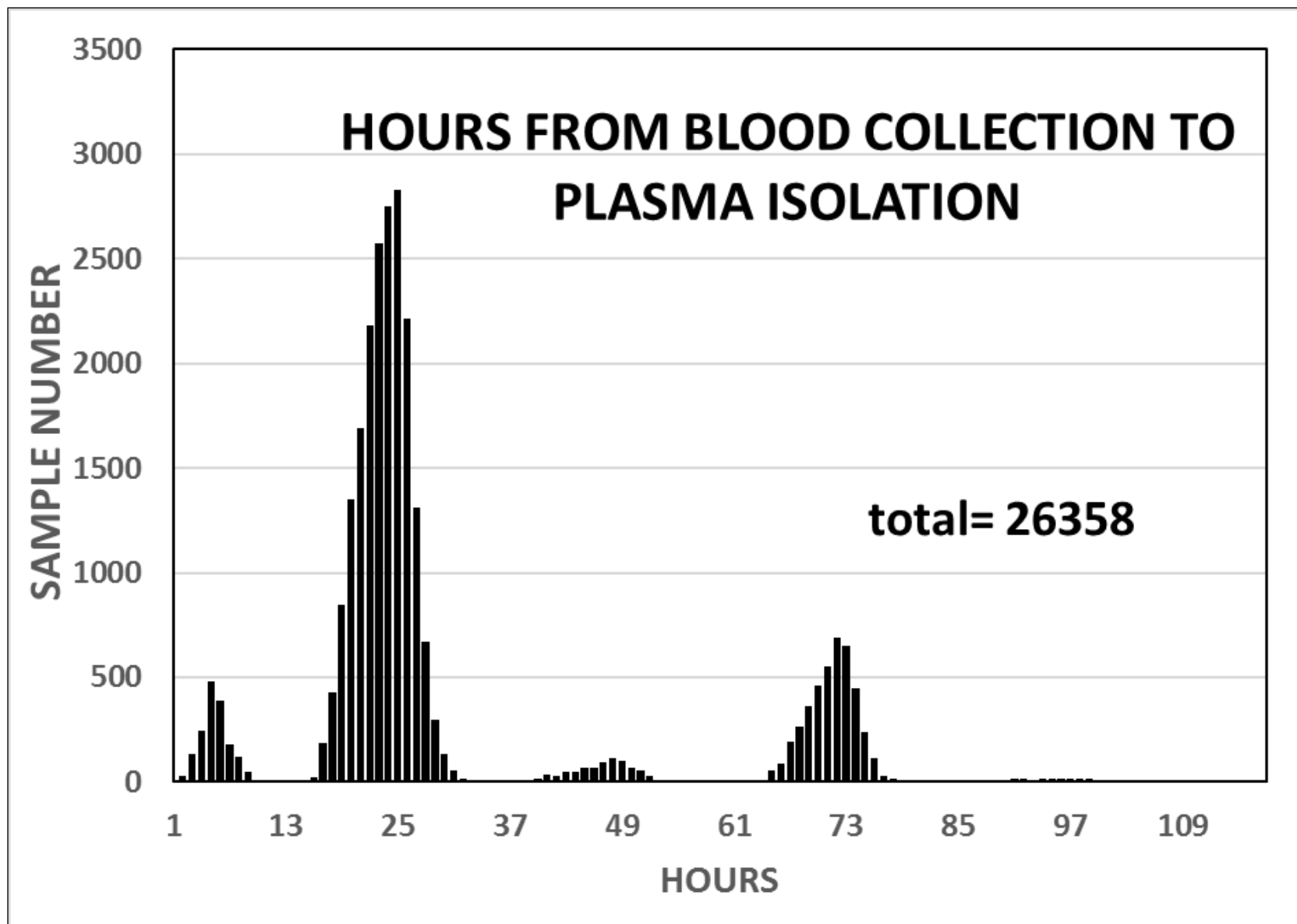

Supplement: Supplemental Figure S3 — Blood to plasma processing times. Vertical bars indicate the number of blood samples processed to plasma at each hourly time point from inception of the Biobank to March 1, 2025, with the 0 time point representing the time of the blood draw. These data include longitudinal samples obtained from patients that were collected and transported in the same manner as initial diagnostic samples. Eighty percent of blood samples (80.3%) were processed to plasma within 29 hours of the patient blood draw, and 96.1% were completed by 72 hours. [file mmc3.pdf]

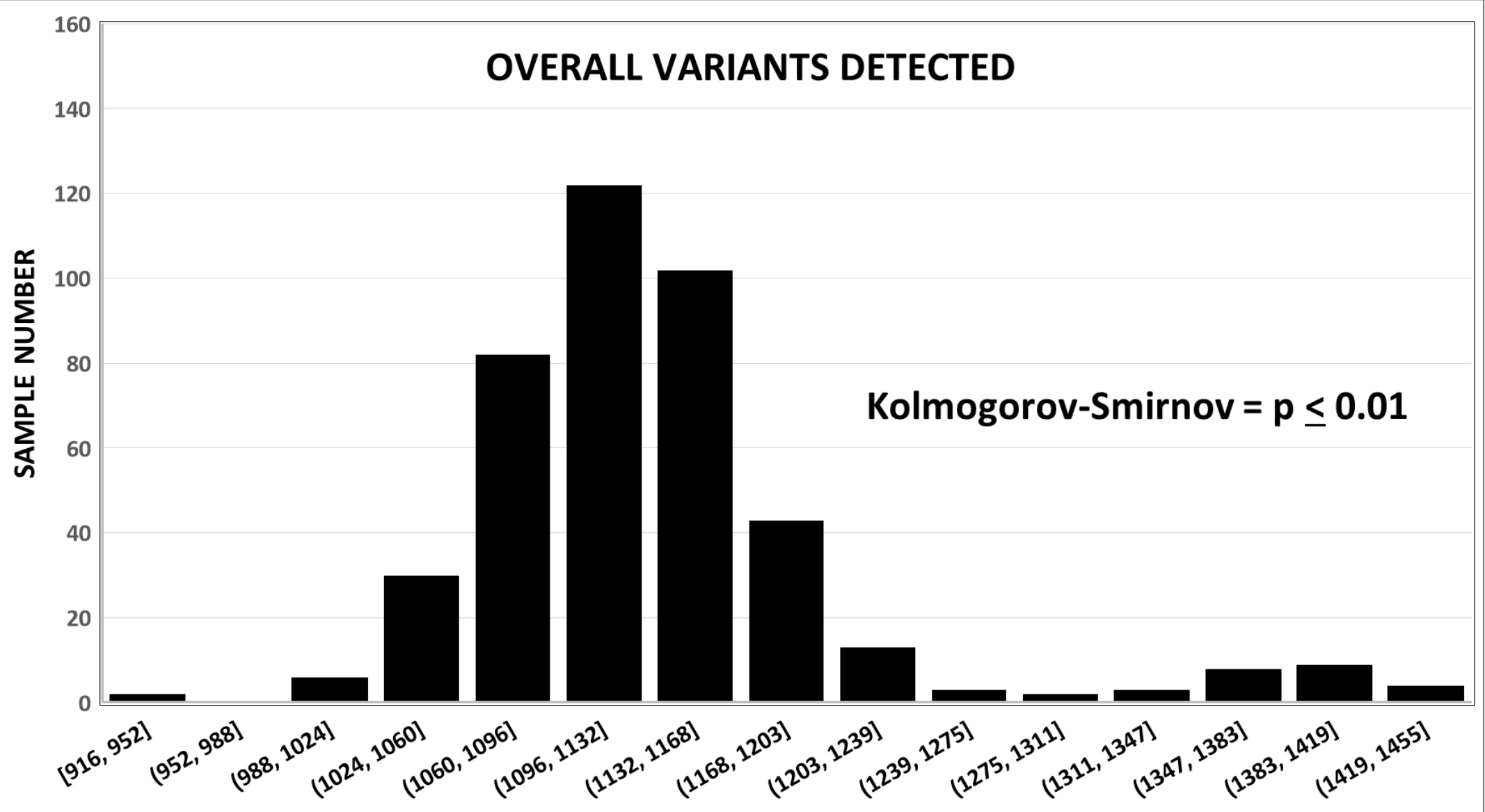

Supplement: Supplemental Figure S4 — Variant detection and distribution in concordance samples. Vertical bars indicate the number of overall variants found in the 430 matched tumor tissue and cell-free DNA samples that underwent sequencing in this study. Individual bins indicate the number of variants detected in samples with each bin increasing in variant number across the range we detected. The circulating tumor DNA variant frequencies and concordance values obtained after sequencing did not conform to a normal distribution (Kolmogorov-Smirnov: P ≤ 0.01) and were natural log transformed for analysis of individual variables. [file mmc4.pdf]
