# Supplementary material for: Cell-Free DNA, Tumor Molecular Concordance, and Clinical Correlates of Patients with Cancer Treated in a Large Community Health Care Network
Source: J Mol Diagn. 2025 Jun 25;27(9):882–98. doi: 10.1016/j.jmoldx.2025.05.007 (PMC12489365; doi:10.1016/j.jmoldx.2025.05.007)
Supplement: Supplemental Table 1 [file mmc5.docx]

**SUPPLEMENTAL TABLE 1.**

|  | **Test Statistic** | **Degrees of Freedom** | **P value** |
| --- | --- | --- | --- |
| **Log-rank test** | **64.8489** | **3** | **0.00001** |

**Comparison: Relationship of cell free DNA Concentration and Overall Survival**

Hazard ratios (cell free DNA Concentration Quartiles)

| **GROUPS** | **HAZARD RATIOS** | **STANDARD ERROR** | **LOWER CONFIDENCE INTERVAL (95%)** | **UPPER CONFIDENCE INTERVAL (95%)** |
| --- | --- | --- | --- | --- |
| **1 vs 2** | **0.4567** | **0.1885** | **0.3218** | **0.6739** |
| **1 vs 3** | **0.3636** | **0.1927** | **0.2492** | **0.5305** |
| **1 vs 4** | **0.1943** | **0.1988** | **0.1316** | **0.2868** |
| **2 vs 1** | **2.1475** | **0.1885** | **1.4840** | **3.1075** |
| **2 vs 3** | **0.7808** | **0.1918** | **0.5361** | **1.1371** |
| **2 vs 4** | **0.4172** | **0.1979** | **0.2831** | **0.6148** |
| **3 vs 1** | **2.7504** | **0.1927** | **1.8851** | **4.0129** |
| **3 vs 2** | **1.2808** | **0.1918** | **0.8794** | **1.8653** |
| **3 vs 4** | **0.5343** | **0.2019** | **0.3597** | **0.7937** |
| **4 vs 1** | **5.1476** | **0.1988** | **3.4866** | **7.5998** |
| **4 vs 2** | **2.3971** | **0.1979** | **1.6265** | **3.5328** |
| **4 vs 3** | **1.8716** | **0.2019** | **1.2600** | **2.7801** |

**Comparison: Relationship of Tumor Stage and Overall Survival**

|  | **Test Statistic** | **Degrees of Freedom** | **P value** |
| --- | --- | --- | --- |
| **Log-rank test** | **44.7353** | **3** | **1.0532^-9^** |

Hazard ratios (Diagnostic Tumor Stage)

| **GROUPS** | **HAZARD RATIOS** | **STANDARD ERROR** | **LOWER CONFIDENCE INTERVAL (95%)** | **UPPER CONFIDENCE INTERVAL (95%)** |
| --- | --- | --- | --- | --- |
| **1 vs 2** | **0.8754** | **0.2252** | **0.5630** | **1.3612** |
| **1 vs 3** | **0.5234** | **0.1991** | **0.3543** | **0.7733** |
| **1 vs 4** | **0.3010** | **0.1953** | **0.2053** | **0.4413** |
| **2 vs 1** | **1.1423** | **0.2252** | **0.7347** | **1.7763** |
| **2 vs 3** | **0.5979** | **0.2131** | **0.3938** | **0.9080** |
| **2 vs 4** | **0.3438** | **0.2095** | **0.2280** | **0.5184** |
| **3 vs 1** | **1.9105** | **0.1991** | **1.2931** | **2.8226** |
| **3 vs 2** | **1.6724** | **0.2131** | **1.1013** | **2.5396** |
| **3 vs 4** | **0.5750** | **0.1812** | **0.4031** | **0.8201** |
| **4 vs 1** | **3.3227** | **0.1953** | **2.2661** | **4.8719** |
| **4 vs 2** | **2.9086** | **0.2095** | **1.9291** | **4.3856** |
| **4 vs 3** | **1.7392** | **0.1812** | **1.2193** | **2.4807** |

**SUPPLEMENTAL TABLE 1: Hazard Ratios for Comparisons of Overall Survival.**

The upper section shows results associated with individual Groups representing cfDNA concentration quartiles depicted in **Figure 5A Kaplan-Meier Curve** demonstrating Hazard Ratios obtained for all quartile group comparisons. Quartile 1 = 0.50 – 3.80 ng/ml, Quartile 2 = 3.83 – 6.35 ng/ml, Quartile 3 = 6.43 – 12.30 ng/ml, Quartile 4 = 12.40 – 1132.90 ng/ml.

The lower section shows results associated with duration of overall survival versus individual tumor stages at time of diagnosis in **Figure 5B Kaplan-Meier Curve** demonstrating Hazard Ratios obtained for all group comparisons. Group 1 = Stage 1, Group 2 = Stage 2, Group 3 = Stage 3, Group 4 = Stage 4.
